# The EXPANDER-1 trial: introduction of the novel Urocross™ Expander System for treatment of lower urinary tract symptoms (LUTS) secondary to benign prostatic hyperplasia (BPH)

**DOI:** 10.1038/s41391-022-00548-z

**Published:** 2022-05-31

**Authors:** Henry H. Woo, Chi-Ping Huang, William J. Huang, Yi-Huei Chang, Chi-Shun Lien, Archil Chkhotua, Dean S. Elterman

**Affiliations:** 1 College of Health and Medicine, Australian National University, Canberra, ACT, Australia and SAN Prostate Centre of Excellence, Sydney Adventist Hospital, Wahroonga, NSW Australia; 2 Department of Urology, China Medical University and Hospital, Taichung, Taiwan; 3 Department of Urology, Taipei Veterans General Hospital; School of Medicine, National Yang Ming Chiao Tung University, Taipei City, Taiwan; 4 Department of Urology, China Medical University and Hospital, Taichung, Taiwan; 5 National Center of Urology, Tbilisi, Georgia; 6 Division of Urology, University Health Network, University of Toronto, Toronto, ON Canada

**Keywords:** Prostatic diseases, Outcomes research

## Abstract

**Purpose:**

To demonstrate the safety and feasibility of the Urocross Expander System (formerly branded as XFLO Expander System), an implantable nitinol tissue expander to trea t patients with lower urinary tract symptoms (LUTS) due to benign prostatic hyperplasia (BPH).

**Materials and methods:**

Men of 50 years or older were eligible to participate in the international, prospective, three-arm, open-label EXPANDER-1 trial if they had a prostate volume between 30 and 80 cc, prostatic urethra length between 20 and 60/80 mm, international prostate symptom score (IPSS) > 13, peak urinary flow (Qmax) < 12 mL/s, post-void residual (PVR) urine volume < 250 mL and quality of life (QoL) score ≥ 3. Patients had pre-assigned implant indwell times (1, 6, and 12 months for Arm-1, Arm-2 and Arm-3 respectively) with follow-up through 6 months (Arm-1) and 3 years (Arm-2 and Arm-3) post-retrieval.

**Results:**

Outcome from treated subjects with their 6-month post-retrieval will be presented in this manuscript, as data collection from longer-term follow-up is ongoing. As of May 24, 2021, 39 and 22 men (mean age: 65), respectively, had implants successfully deployed and retrieved without any complications. No cases of implant encrustation were observed. Device- and procedure-related adverse events were predominantly mild to moderate in severity. Three SAEs were reported. Only one patient required catheterization post-implant for more than three days. Improvements in clinical parameters such as IPSS, QoL, PVR and Qmax as well as sexual function were observed.

**Conclusions:**

Preliminary results demonstrate that the Urocross Expander System is a feasible and safe procedure for treating BPH/LUTS. A strong signal of efficacy justifies further evaluation of this PRostatic Urethral Expansion (PURE) procedure. Negative features of earlier generations of prostatic implants such as biocompatibility, migrations and encrustation have possibly been overcome.

## Introduction

Benign Prostatic Hyperplasia (BPH) is a progressive condition commonly associated with bladder outflow obstruction and lower urinary tract symptoms (LUTS) [1].

Surgery, specifically transurethral resection of the prostate (TURP), was once the cornerstone for the treatment of BPH/LUTS after drug therapy fails. However, because TURP has the potential for significant morbidity [2], a plethora of minimally invasive surgical therapies (MISTs) have been developed as treatment alternatives. Among these are mechanical techniques, such as the prostatic urethral lift (UroLift System, NeoTract/Teleflex, Pleasanton, California, USA [3, 4]) and the temporary implantable nitinol device (iTind, Medi-Tate Ltd, Hadera, Israel/Olympus Corp, Tokyo, Japan [5–7]), as well as ablative techniques, such as water vapor thermal therapy (Rezum, NxThera Inc., Maple Grove, Minnesota, USA/Boston Scientific Corp, Marlborough, Massachusetts, USA [8]) and Aquablation (Aquabeam System, Procept BioRobotics, Redwood Shores, California, USA [9]). To be considered successful, a minimally invasive BPH treatment should provide (a) rapid relief from symptoms, (b) durable symptom relief, (c) minimal possibility of adverse events, (d) and minimal anesthesia requirements allowing for treatment in the outpatient setting [10].

The Expander System (Prodeon Medical, Inc. (PMI), Sunnyvale, California, USA) is a MIST that has been developed for use with a flexible cystoscope and includes an implantable tissue expander (the Expander implant or implant) and delivery system (handle and delivery catheter) designed for placement of the implant in the prostatic urethra and its retrieval after implantation for a minimum of one month and up to 12 months. The implant is a new nitinol device with a quadrant, strut-like design developed with the aim to expand and reshape the prostatic urethra, through gentle mechanical tissue retraction and relieve urinary outflow obstruction symptoms and LUTS. Early results from the first patients were promising with most patients reporting rapid symptom relief and minimal complications [11].

The objective of the EXPANDER-1 trial was to demonstrate the safety and feasibility of the Expander System procedure to treat patients with BPH/LUTS.

## Materials and methods

### Investigational devices

The Expander System consists of a sterile, single-use nitinol tissue expander implant preloaded in a delivery catheter. Each delivery catheter is designed to advance through the working channel of a flexible cystoscope. Using the handle of the delivery system, the controlled deployment of the implant in the prostatic urethra is performed under direct cystoscopic visualization.

The implant has four arms connected by two hubs (Supplementary Fig. 2). The quadrant, strut-like design aims to gently retract and reshape the obstructing prostatic tissue lobes. The implant is retrieved using the sterile single-use purpose-designed Retrieval Sheath (Prodeon Medical, Inc., USA) in conjunction with commercially available and compatible flexible cystoscopes and graspers. The Retrieval Sheath has been designed to accommodate the passage of a flexible cystoscope while also having sufficient flexibility and length to reach the prostatic urethra.

This trial evaluated a first-generation Expander System and was subsequently amended to include evaluation of a second-generation Expander System. Both systems use identical implants, with an enhanced delivery catheter from the first generation to the second. The first-generation Expander System was only available as a small implant length (20 mm long × 18 mm diameter); however, the second-generation Expander System included an additional option to use a larger implant.

### Implantation and retrieval procedures

For implantation, a flexible cystoscope is used to measure the prostatic urethral length from bladder neck to the verumontanum. For second-generation-device patients, small and large implants are deployed for prostatic urethral lengths of 20–30 mm and >30 mm, respectively. The delivery catheter is first inserted through the port of the flexible cystoscope. Then the delivery handle is locked to the cystoscope. An irrigation line is connected to the handle to allow continuous irrigation during the procedure. Adjustments to the position of the tip of the delivery catheter can be made on the delivery handle. Using standard urology technique, the scope and catheter are inserted to the prostatic urethra. Once the catheter tip is positioned at the level of the verumontanum, the slider on the delivery handle is slowly advanced allowing the deployment of the implant between the verumontanum and bladder neck. The implant arms expand outwardly, retract the prostatic tissue and widen the prostatic urethra lumen (Supplementary Fig. 3). Finally, the delivery catheter and cystoscope are removed.

For implant retrieval, the flexible cystoscope is inserted through the lumen of the Retrieval Sheath. Both the Retrieval Sheath and cystoscope are introduced as a single unit and under direct visualization, the distal hub of the implant is identified. A rat-tooth grasper is then advanced through the instrument (working) channel of the cystoscope and the implant hub is secured inside the jaws of the grasper. The Retrieval Sheath is carefully advanced to bring it within the cystoscope’s field of view in order to capture the implant hub. While holding the Retrieval Sheath, the cystoscope is withdrawn along with the grasper and implant into the Retrieval Sheath lumen. Once the collapsed implant is inside the Retrieval Sheath, all components (implant, Retrieval Sheath, cystoscope and grasper) are removed from the urethra as one unit.

Procedures were performed under topical anesthesia, mild intravenous sedation, or general anesthesia in a cystoscopy suite or operating room.

### Trial design

This prospective, non-randomized, three-arm, multi-center, open-label, clinical trial evaluates the safety and feasibility of the Expander System in patients with LUTS due to BPH. This trial is being conducted at 4 centers in Taiwan, Canada and Australia.

The protocol was approved by the ethics committees/institutional review boards at all enrolling centers (ClinicalTrials.gov: NCT03758222) and all enrolled patients signed an informed consent.

Inclusion and exclusion criteria are summarized in Supplementary Table 3. All patients agreed to a stable BPH medication regimen throughout the trial prior to the implant procedure (i.e., the drug or drug dose would not be altered or discontinued, unless clinically indicated). Patients were excluded if the investigator felt that significant medical comorbidity or previous surgery could impact clinical trial participation or confound trial outcomes.

In Arm 1, the implant indwell time was 1 month and post-retrieval follow-up at 6 months. Safety and effectiveness data were assessed at follow-up visits after 2 weeks and 1-month post-implantation, and up to 6 months following implant retrieval [11]. In Arms 2 and 3, the implant indwell time was 6 and 12 months, respectively, then retrieved with a follow-up of the patients at 2 weeks, 1-, 3-, 6-, and 9 months, then yearly up to 3 years following implant retrieval. While the devices are implanted, patients are evaluated at 2 weeks, 1-, 3-, and 6 months for Arm 2 and at 2 weeks, 1-, 3-, 6-, 9- and 12 months for Arm 3. Enrollment has been completed in the study with 45 subjects’ treatment. Retrieval (in Arm 3) as well as follow-up in (Arm-2 and Arm-3) are in progress.

For safety purposes, the trial design consisted of a staged approach in which 5 patients were initially enrolled and treated in Arm 1 with the shorter indwell time of 1 month. Once data from Arm 1 was reviewed and use of the device was assessed as safe by an independent data monitoring committee (DMC), Arm 2 was initiated, followed by Arm 3 with a longer indwell time of 6 and 12 months, respectively.

The primary safety endpoint is the cumulative rate of device- and/or procedure-related adverse events from device implantation to 6 months following implant retrieval for each treatment arm. Other safety endpoints included incidence of repeat invasive treatment for LUTS (defined as any BPH-related serious adverse event (SAE) resulting in surgical intervention including minimally invasive procedures), and freedom from unanticipated adverse device effects. Acute and long-term implantation and post-retrieval effectiveness was evaluated using the following questionnaires and instruments: IPSS, uroflowmetry, post-void residual (PVR) urine volume, quality of life (QoL) score, Sexual Health Inventory for Men (SHIM), Male Sexual Health Questionnaire for Ejaculatory Dysfunction (MSHQ-EjD), Michigan Incontinence Symptom Index (M-ISI) questionnaire. Additionally, safety and effectiveness were further assessed by the technical success of placement and retrieval. All safety and effectiveness assessments, including adverse events and medication review, were performed during the post-implant and post-retrieval follow-up period.

Patients were also scheduled to undergo follow-up cystoscopies at 1-month post-retrieval (Arm 1), 9 (or 12) and 36 months post-retrieval (Arm 2), and 6 (or 9) months post-implant and 9 (or 12) and 36 months post-retrieval (Arm 3). Also, at 3 months post-implant, an ultrasound was to be performed to assess the location of the implant during the indwell period (Arm 3).

### Statistical methods

Sample size was calculated using the simple asymptotic method. Data cut-off for this evaluation was on 24 May 2021. For continuous variables, data were expressed as mean and standard deviation (mean ± SD); for categorical variables, data were expressed as frequency counts and percentages. All endpoints were summarized by each treatment arm and overall treatment arms. Changes from baseline were calculated for each patient using their post-baseline value minus baseline value. Results were described for the full analysis population, consisting of enrolled patients who had met all eligibility criteria, had a successful implant procedure and had at least one follow-up assessment completed. SAS® software version 9.4 or higher was used for statistical analysis.

## Results

A total of 39 men (mean age of 65 years) were treated between 09 November 2018 and 24 May 2021: 16 and 23 patients with the first- and second-generation Expander Systems, respectively (32 small and 7 large implants). During the trial, 33.3% of patients received new BPH medication that included 5ARI, alpha blockers, and antimuscarinics after baseline due mainly to reported AEs.

The initial patients were treated in Arm 1 (*N* = 5) under the first-in-human protocol. Upon review by an independent DMC, it was then decided to proceed with Arm 2 (*N* = 15) and Arm 3 (*N* = 19) under the revised feasibility protocol. Patient’s disposition can be found in Supplementary Fig. 4. In Arm 1, one patient was lost to follow-up 3 months after the 3- months post-retrieval follow-up visit. In Arm 2, one patient who was lost to follow-up, did not return for his retrieval procedure due to major complications associated with pre-existing respiratory disease, and another patient was treated but requested to be withdrawn 2 days post-procedure at which timepoint his implant was retrieved. Another patient withdrew from the trial after his 1-month post-retrieval follow-up visit due to skin cancer recurrence requiring treatment. All Arm 3 patients remain in the trial and are in the follow-up phases while recruitment is ongoing at time of data cut-off (Supplementary Table 4).

Prior to hospital discharge following both the device implantation and retrieval, all patients were able to urinate. One patient required catheterization post-implant for more than three days.

### Safety

All implants were successfully deployed in the prostatic urethra without any SAEs or complications. Four device deficiencies, of which two were attributed to scope compatibility, were reported during implantation without an associated adverse event. Of the 39 patients who had been implanted, 22 had their implants successfully retrieved (including the premature retrieval due to patient withdrawal 2 days post-procedure) without any complications or unanticipated SAEs. During retrieval, two device deficiencies, one for the implant and one for the retrieval sheath, both due to user error, were reported. No cases of implant encrustation were observed in any patient at the time of cystoscopic follow-up examinations or following any implant retrieval.

A Clavien-Dindo Classification summary of adjudicated device- and procedure-related adverse events, predominantly mild to moderate in severity, is shown in Table 1. To date, there were 3 SAEs reported, but only two were related to the device and/or the procedure as adjudicated by the DMC. Both related events required a repeat invasive treatment. The first related SAE was an asymptomatic migration of the implant into the bladder identified at the 6-month follow-up. Using a grasping forceps, repositioning of the implant was successful and uneventful. No further events were reported as related to this device migration. The second related SAE was hematuria, which occurred 4 days post-implant and required intervention for blood clot removal. Blood clot formation was likely due to an ASA-related coagulopathy, as the patient was on aspirin therapy at the time of the procedure. The SAE was adjudicated as being device related. The third SAE was deterioration of chronic obstructive pulmonary disease due to pneumonia requiring hospitalization and was adjudicated as not related to the device or the procedure. There were no unanticipated adverse device effects.

**Table 1:** Safety outcome – Clavien-Dindo classification of device- and procedure-related adverse events^a^ (DMC Adjudicated).

Adverse event	Number of events	Number of patients (%)	Grade I	Grade II	Grade IIIa	Grade IIIb	Grade IV	Grade V
Haematuria	8	7 (17.9%)	7	0	0	1	0	0
Micturition Urgency	6	5 (12.8%)	4	2	0	0	0	0
Dysuria	4	4 (10.3%)	3	1	0	0	0	0
Incontinence	4	4 (10.3%)	3	1	0	0	0	0
Nocturia	3	3 (7.7%)	0	3	0	0	0	0
Pollakiuria	3	3 (7.7%)	2	1	0	0	0	0
Procedural pain	2	2 (5.1%)	2	0	0	0	0	0
Urine flow decreased	2	2 (5.1%)	1	1	0	0	0	0
Bladder catheter temporary	1	1 (2.6%)	0	1	0	0	0	0
Device dislocation/Migration	1	1 (2.6%)	0	0	0	1	0	0
Lower urinary tract symptoms	1	1 (2.6%)	0	1	0	0	0	0
Pelvic pain	1	1 (2.6%)	1	0	0	0	0	0
Syncope	1	1 (2.6%)	1	0	0	0	0	0
Urethral Stenosis/Stricture	1	1 (2.6%)	0	0	1	0	0	0
Urethritis noninfective/Irritation	1	1 (2.6%)	0	1	0	0	0	0
Urinary retention	1	1 (2.6%)	0	1	0	0	0	0
Total	40	22 (56.4%)	24	13	1	2	0	0

Overall complication rates and complications graded by using the Clavien-Dindo classification and adjudicated by DMC.

^a^Ref. [16].

### Effectiveness

**Fig. 1:**
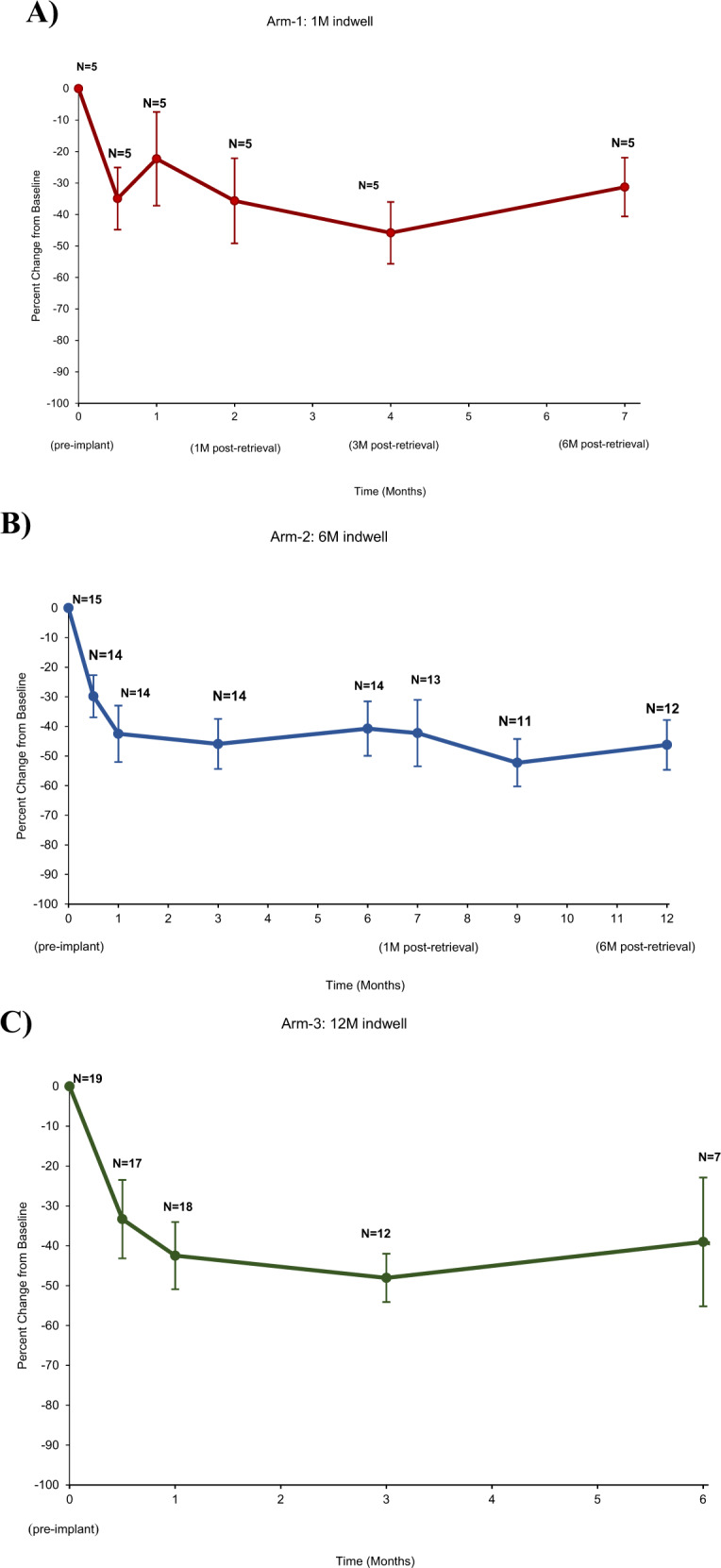
Percent change from baseline in AUA/IPSS total score-full analysis set. **A** Percent change from baseline in AUA/IPSS total score-full analysis set. Percent change from baseline (calculated as post-baseline value – baseline value) for AUA/IPSS in the Arm-1 cohort. For the Arm-1 cohort, patients experienced a 1-month indwell duration and a 6-month post-retrieval. Error bars indicate SEM. **B** Percent change from baseline in AUA/IPSS total score-full analysis set. Percent change from baseline (calculated as post-baseline value – baseline value) for AUA/IPSS in Arm-2 cohort. For the Arm-2 cohort, patients experienced a 6-month indwell duration and a 6-month post-retrieval. Error bars indicate SEM. **C** Percent change from baseline in AUA/IPSS total score-full analysis set. Percent change from baseline (calculated as post-baseline value – baseline value) for AUA/IPSS in Arm-3 cohort. For the Arm-3 cohort, patients experienced a 12-month indwell duration. Error bars indicate SEM.

Effectiveness data for IPSS is summarized in Table 2 and Fig. 1. Uroflowmetry, QoL and PVR data are summarized in the Supplementary Appendix Tables. There was no observation of significant deterioration of sexual function and ejaculatory function as assessed by the SHIM and MSHQ-EjD questionnaires. Overall, severity and bother of patients’ incontinence tended to slightly improve as shown by decreasing M-ISI total and bother scores at 6 months post-retrieval.

**Table 2:** Total IPSS scores – full analysis set.

		Post-implant							Post-retrieval		
		2 w	1 M	3 M	6 M	9 M	12 M		1 M	3 M	6 M
Arm 1 (*N* = 5)	*N*	5	5	NA				Retrieval	5	5	4
	Baseline	24.0 (5.8)	24.00 (5.8)						24.0 (5.8)	24.0 (5.8)	24.0 (5.8)
	Follow-up	16.2 (8.0)	18.2 (8.1)						15.6 (9.2)	12.4 (3.8)	18.3 (6.9)
	Change	−7.80 (5.0)	−5.8 (7.3)						−8.4 (7.4)	−11.6 (6.0)	−8.0 (5.4)
	% Change	−34.9 (22.1)	−22.3 (33.3)						−35.7 (30.2)	−45.8 (21.9)	−31.3 (21.0)
Arm 2 (*N* = 15)	*N*	14	14	14	14	NA			13	11	12
	Baseline	23.9 (5.0)	23.9 (5.0)	23.9 (5.0)	23.9 (5.0)				23.9 (5.0)	23.9 (5.0)	23.9 (5.0)
	Follow-up	17.4 (8.5)	13.9 (9.4)	12.6 (7.9)	13.9 (8.5)				14.4 (10.0)	12.2 (7.1)	13.8 (7.9)
	Change	−6.4 (6.2)	−9.9 (9.2)	−11.2 (9.1)	−9.9 (8.9)				−10.1 (9.7)	−12.8 (6.7)	−10.9 (6.7)
	% Change	−29.8 (29.7)	−42.5 (35.7)	−45.9 (31.6)	−40.7 (34.5)				−42.2 (40.4)	−52.3 (26.6)	−46.2 (29.2)
Arm 3 (*N* = 19)	*N*	17	18	12	7	NA			NA		
	Baseline	22.4 (5.4)	22.4 (5.4)	22.4 (5.4)	22.4 (5.4)						
	Follow-up	14.3 (7.9)	12.8 (7.1)	12.0 (6.6)	11.6 (7.9)						
	Change	−7.9 (8.9)	−10.1 (7.9)	−10.6 (4.9)	−8.1 (9.4)						
	% Change	−33.3 (40.5)	−42.2 (36.6)	−48.1 (21.0)	−39.0 (42.7)						
Total (*N* = 39)	*N*	36	37	26	21	NA			18	16	16
	Baseline	23.2 (5.2)	23.2 (5.2)	23.2 (5.2)	23.2 (5.2)				23.2 (5.2)	23.2 (5.2)	23.2 (5.2)
	Follow-up	15.8 (8.0)	13.9 (8.1)	12.3 (7.2)	13.1 (8.2)				14.7 (9.5)	12.3 (6.1)	14.9 (7.7)
	Change	−7.3 (7.3)	−9.4 (8.2)	−10.9 (7.3)	−9.3 (8.8)				−9.7 (9.0)	−12.4 (6.3)	−10.2 (6.3)
	% Change	−32.2 (33.7)	−39.6 (35.5)	−46.9 (26.8)	−40.2 (36.4)				−40.4 (37.1)	−50.3 (24.7)	−42.5 (27.5)

The total IPSS scores is calculated as the sum of all responses.

Values are presented as mean (SD).

## Discussion

This is the first clinical report of the Expander System as a MIST for the treatment of BPH/LUTS. This technology is a novel treatment designed to be implanted using a flexible cystoscope. The implant is designed to reshape the prostatic tissue and to disobstruct the prostatic urethral lumen without heating, burning, ablating, cutting or removing any prostate tissue.

To date, the Expander implant was associated with successful deployments and retrievals in the current clinical trial and has met the co-primary objective of safety and feasibility. The Expander System has also been confirmed as minimally invasive and meeting the co-primary objective for safety. The proposed mechanism of action by PURE using the new nitinol Expander implant appears concordant with the clinical effectiveness observed in these preliminary results.

Numerous prostatic stent devices have been trialed over the past 40 years, but few remain in clinical use. Major problems with early generation stents included device migration and encrustation [12]. These early stents often had poor biocompatibility in the urinary environment and this, in combination with encrustation, created serious technical challenges because of obstruction within the lumen of the stent or with their removal. Accordingly, an important finding was the absence of any encrustation or stone formation on the implant during both follow-up surveillance and retrieval cystoscopy examinations.

One patient experienced implant migration into the bladder, which was an unexpected finding due to the nature of the device expanding into the prostatic urethra with expected holding of its position. A careful review of device deployment videos confirmed that placement had been sub-optimally too proximal, potentially providing a reason for this implant migration. The solitary case of implant migration equates to an incidence of 2.6% and is not considered a major concern for the overall outcome of the trial.

Reports of clinical parameters such as IPSS, QoL, PVR, Qmax and sexual function were not amongst the primary objectives of this trial, however, there were observed improvements similar to those of other MISTs [13] that would certainly justify continued evaluation of this PURE approach for treating BPH/LUTS.

To date, increased indwell time is associated with having a higher risk of implant migration. Our current data showed a reduction of symptoms during the 6-month indwell time and this duration has been shown to have satisfactory outcome data with little risk of migration. As such, the 6-month indwell duration will be used in future studies.

Potential benefits of using the Expander System include the ability to be deployed using a flexible cystoscope without the requirement for additional equipment.

This trial is limited by the variations in treatment approaches reported in this trial including differing implant dwell times, changing from first- to second-generation delivery system and associated inclusion of a larger implant option and limited clinical outcomes data past 6-month post-implant retrieval. These limitations will largely be overcome in future reports as larger series are reported.

## Conclusion

Preliminary clinical results with a novel PURE procedure, using the Expander System, have met the trial objectives in terms of feasibility and safety.

To date, the Expander System provides a signal of efficacy similar to that observed in other early-stage MIST trials [14, 15]. This, in addition to a favorable safety profile, justifies further evaluation of a PURE approach for treating BPH/LUTS. These results also suggest that the negative features of earlier generations of prostatic stents such as biocompatibility, migrations and encrustation have been overcome.

## Supplementary information

Supplementary Legends

Supplementary Figure 2

Supplementary Figure 3

Supplementary Figure 4

Supplementary Table 3

Supplementary Table 4

## Data Availability

The data that support the findings of this study are available from Prodeon Medical, Inc., but restrictions apply to the availability of these data and so are not publicly available. Data are however available from the authors upon reasonable request and with permission of PMI.
