# Supplementary material for: The EXPANDER-1 trial: introduction of the novel Urocross™ Expander System for treatment of lower urinary tract symptoms (LUTS) secondary to benign prostatic hyperplasia (BPH)
Source: Prostate Cancer Prostatic Dis. 2022 May 31;25(3):576–82. doi: 10.1038/s41391-022-00548-z (PMC9385491; doi:10.1038/s41391-022-00548-z)
Supplement: Supplementary file 1 — Supplementary Legends [file 41391_2022_548_MOESM1_ESM.docx]

Supplementary Figure Legends

**Supplementary Figure 2: Dimensions for the Two Nitinol Implants Available for the Expander System.** The Urocross Implant is manufactured from nitinol and is comprised of two hubs and four outward expanding arms. The unconstrained dimensions of the available sizes are 18 mm x 20 mm and 25 mm x 30 mm (diameter x length) based on the patients’ indicated prostatic urethra length.

**Supplementary Figure 3A: Schematic Illustration of the Procedural Steps of the Implant.** Stepwise depiction of Urocross Implant deployment. (1) Insert catheter into the flexible cystoscope. (2) Connect handle luer to cystoscope. (3) Connect irrigation to the delivery system. (4) Deploy the Expander. (5) Remove cystoscope catheter as a system.

**Supplementary Figure 3B: Schematic Illustration of the Procedural Steps of the Implant Retrieval.** Stepwise depiction of Urocross Implant retrieval. (1) Advance the cystoscope and Retrieval Sheath as a system. (2) Advance the grasper through the center port of the irrigation valve and the working channel of the cystoscope then secure the Expander. (3) Advance the sheath within view of the cystoscope. (4) Retract the Expander and cystoscope into the Sheath as a system. (5) Remove the system from the urethra.

**Supplementary Figure 3C: Schematic Illustration of the Prostatic Lumen.** The prostatic lumen (1) before Urocross Implant deployment and (2) after Urocross Implant deployment.

**Supplementary Figure 4: Patient Flow Diagram.** Consort diagram of patient enrollment, allocations, treatment, and follow up throughout the study.

**Supplementary Table Legends**

**Supplementary Table 3: Inclusion and Exclusion Criteria.** Criteria for study inclusion or exclusion.

**Supplementary Table 4: Baseline demographics and disease characteristics.** General characteristics of Arm-1, Arm-2, and Arm-3 study participants.
