# Supplementary material for: The EXPANDER-1 trial: introduction of the novel Urocross™ Expander System for treatment of lower urinary tract symptoms (LUTS) secondary to benign prostatic hyperplasia (BPH)
Source: Prostate Cancer Prostatic Dis. 2022 May 31;25(3):576–82. doi: 10.1038/s41391-022-00548-z (PMC9385491; doi:10.1038/s41391-022-00548-z)

**Supplementary Figure**

**Figure 2 Dimensions for the Two Nitinol Implants Available for the Expander System**

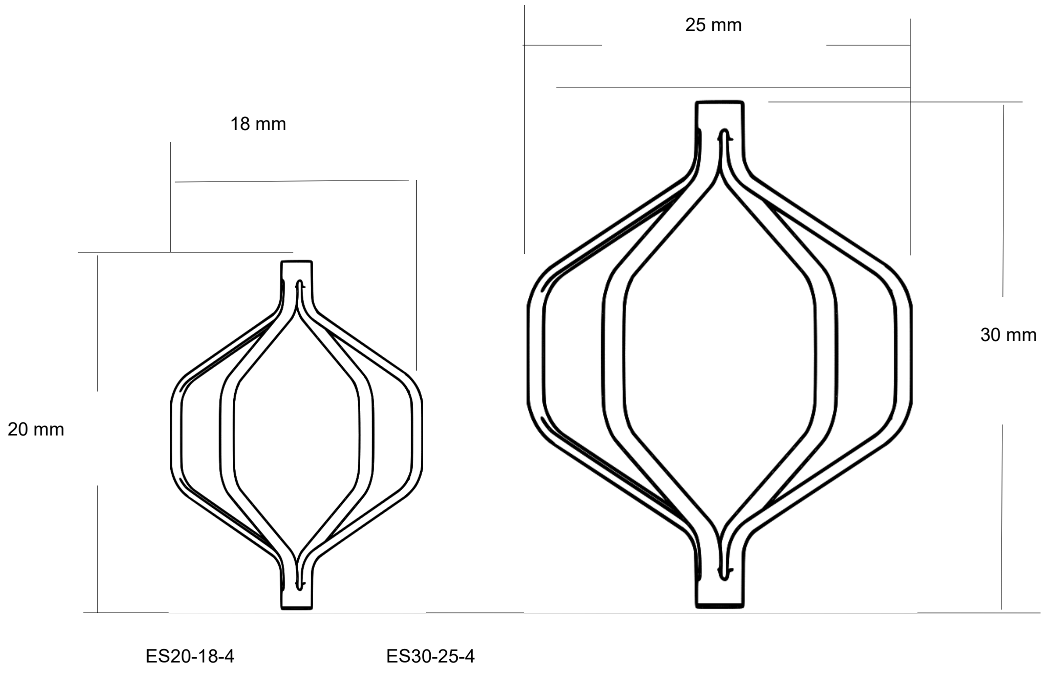

Supplement: Supplementary file 2 — Supplementary Figure 2 [file 41391_2022_548_MOESM2_ESM.pdf]
