# Supplementary material for: The EXPANDER-1 trial: introduction of the novel Urocross™ Expander System for treatment of lower urinary tract symptoms (LUTS) secondary to benign prostatic hyperplasia (BPH)
Source: Prostate Cancer Prostatic Dis. 2022 May 31;25(3):576–82. doi: 10.1038/s41391-022-00548-z (PMC9385491; doi:10.1038/s41391-022-00548-z)

Supplementary Figure 3: Schematic Illustration of the Procedural Steps Before and After Placement of the Implant in the Prostatic Urethral Lumen

A. Schematic Illustration of the Procedural Steps of the Implant

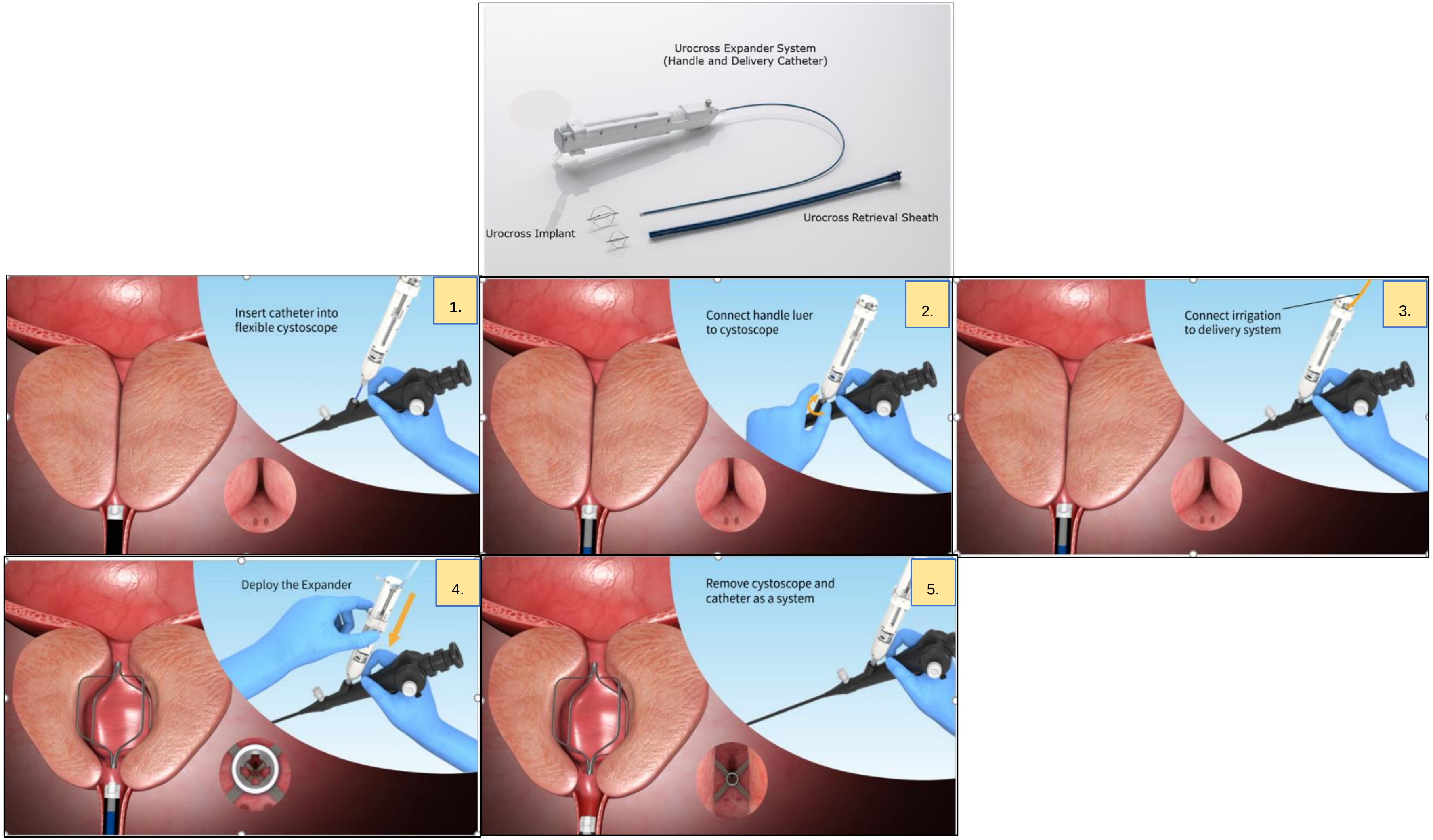

B. Schematic Illustration of the Procedural Steps of the Implant Retrieval

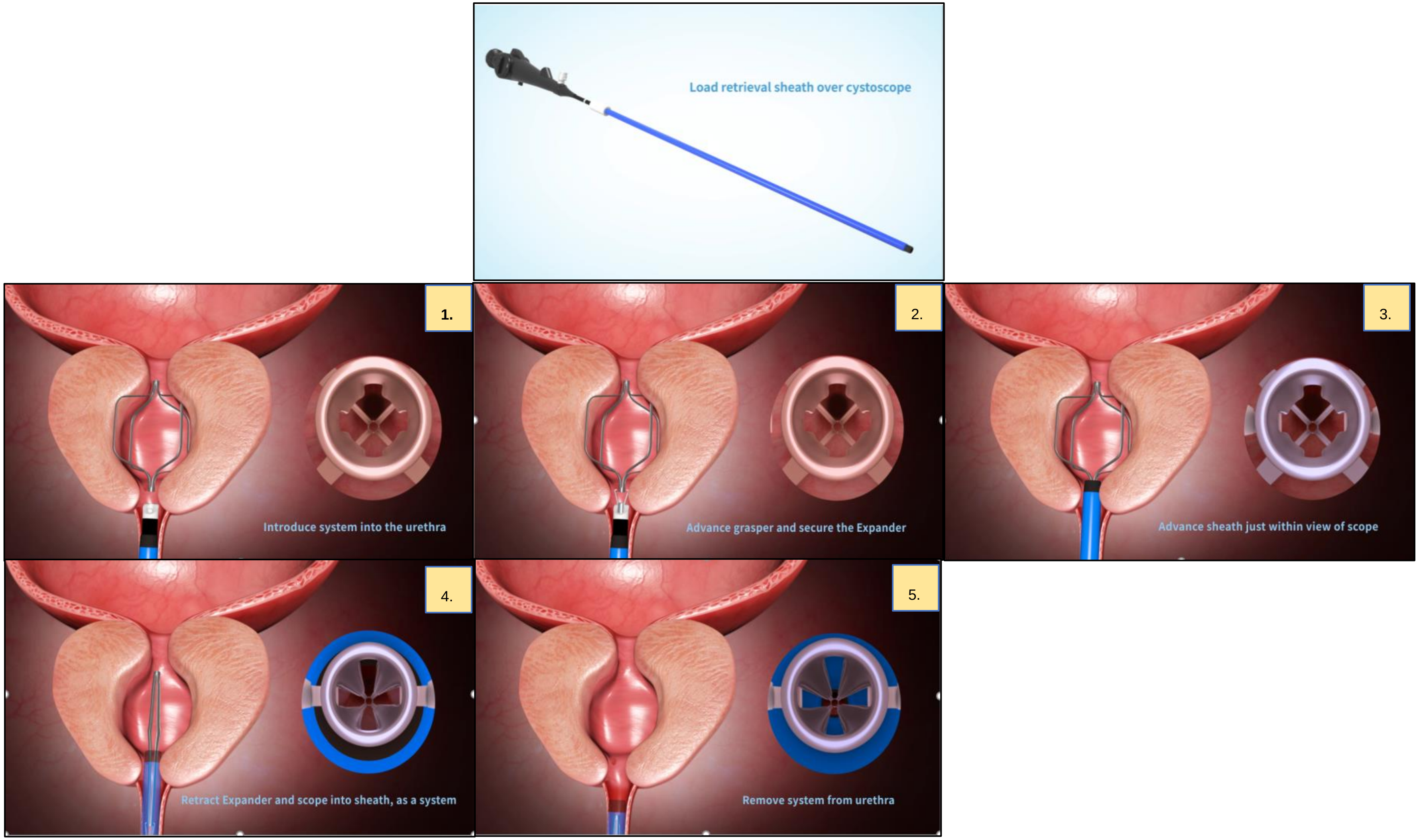

C. Schematic Illustration of the Prostatic Urethral Lumen Before and After Placement of the Implant

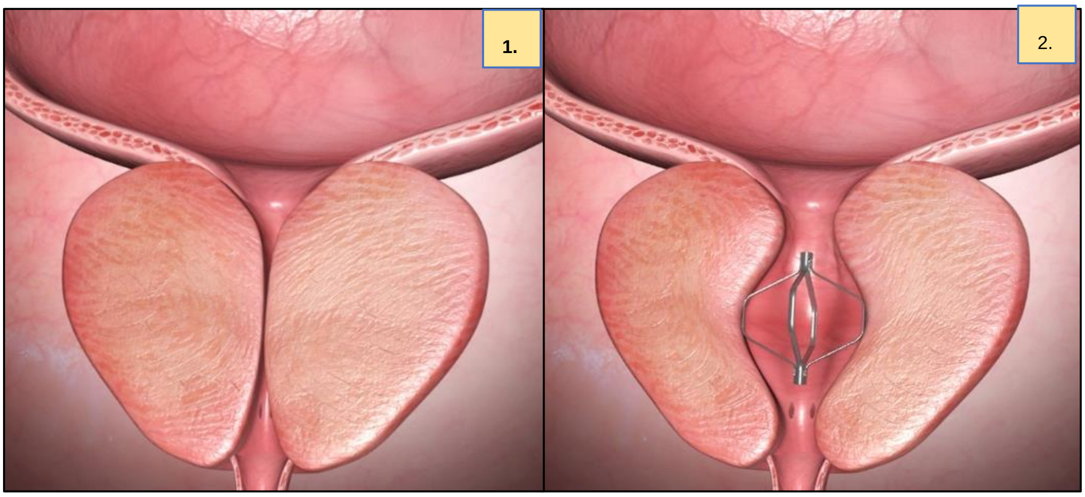

Supplement: Supplementary file 3 — Supplementary Figure 3 [file 41391_2022_548_MOESM3_ESM.pdf]
