# Supplementary material for: The EXPANDER-1 trial: introduction of the novel Urocross™ Expander System for treatment of lower urinary tract symptoms (LUTS) secondary to benign prostatic hyperplasia (BPH)
Source: Prostate Cancer Prostatic Dis. 2022 May 31;25(3):576–82. doi: 10.1038/s41391-022-00548-z (PMC9385491; doi:10.1038/s41391-022-00548-z)
Supplement: Supplementary file 6 — Supplementary Table 4 [file 41391_2022_548_MOESM6_ESM.pdf]

Baseline Demographics and Disease Characteristics

Characteristic	Arm 1 1M Indwell (N=5)	Arm 2 6M Indwell (N=15)	Arm 3 12M Indwell (N=19)	Overall (N=39)
Age (years), Mean (SD)	69.2 (4.97)	64.5 (7.02)	65.0 (6.06)	65.3 (6.36)
BMI (kg/m^2), Mean (SD)	-	24.9 (3.23)[a]	25.8 (2.86)	25.5 (2.95)[b]
Qmax (mL/s), Mean (SD)	8.4 (1.72)	9.3 (2.32)	8.4 (1.87)	8.7 (2.04)
PVR volume (mL), Mean (SD)	162.4 (58.71)	103.2 (68.00)	91.2 (87.38)	104.9 (78.83)
PSA (ng/mL), Mean (SD)	5.7 (5.88)	2.4 (1.68)	1.9 (1.58)	2.6 (2.71)
Creatinine (mg/dL), Mean (SD)	0.9 (0.20)	0.9 (0.16)	1.0 (0.28)	1.0 (0.23)
Prostate volume (mL), Mean (SD)	49.2 (15.79)	46.7 (13.77)	45.6 (12.97)	46.5 (13.31)
IPSS total score, Mean (SD)	24.00 (5.788)	23.87 (5.027)	22.37 (5.418)	23.15 (5.229)
IPSS voiding score, Mean (SD)	13.20 (5.805)	13.80 (4.039)	13.95 (3.628)	13.79 (3.981)
IPSS storage score, Mean (SD)	10.80 (1.643)	10.07 (2.251)	8.42 (2.735)	9.36 (2.570)
QoL score, Mean (SD)	5.0 (0.71)	4.9 (1.06)	4.6 (0.90)	4.7 (0.94)
M-ISI score, Total Domain, Mean (SD)	7.8 (4.02)	5.1 (5.22)	5.2 (3.92)	5.5 (4.45)
M-ISI score, Bother Domain, Mean (SD)	3.4 (2.19)	1.9 (2.07)	1.6 (1.92)	1.9 (2.04)
SHIM score, Mean (SD)	15.8 (10.55)	12.5 (9.05)	13.8 (8.47)	13.6 (8.77)
MSHQ-EjD score, Mean (SD)	8.4 (4.39)	6.5 (3.27)	7.2 (3.52)[c]	7.1 (3.49)[d]
Patients on at least 1 BPH medication, n (%)	2 (40.0%)	10 (66.7%)	14 (73.7%)	26 (66.7%)
5-ARI	0	2 (13.3%)	5 (26.3%)	7 (17.9%)
Alpha-blocker	2 (40.0%)	7 (46.7%)	11 (57.9%)	20 (51.3%)
Muscarinic receptor antagonist	0	3 (20.0%)	0	3 (7.7%)
Other	0	0	1 (5.3%)	1 (2.6%)

[a] N=9

[b] N=28

[c] N=18

[d] N=38
